# Maternal Morbidity and Disability and Their Consequences: Neglected Agenda in Maternal Health

**DOI:** 10.3329/jhpn.v30i2.11294

**Published:** 2012-06

**Authors:** Marge Koblinsky, Mahbub Elahi Chowdhury, Allisyn Moran, Carine Ronsmans

**Affiliations:** ^1^icddr,b, GPO Box 128, Dhaka 1000, Bangladesh; ^2^John Snow Inc., Arlington, Virginia, USA; ^3^Saving Newborn Lives, Save the Children Federation, Washington, DC, USA; ^4^London School of Hygiene & Tropical Medicine, London, UK

## INTRODUCTION

Women's ill-health and its consequences are poorly defined. Despite women living longer than men, their lives are not necessarily healthy, according to the 2009 Women and Health Report of the World Health Organization (WHO) (1). One condition that impacts only women and may contribute to continued ill-health is pregnancy and childbirth. Whereas the appropriate use of skilled birth attendance with supportive emergency obstetric care can reduce health risks during pregnancy and childbirth, there are negative consequences of maternal ill-health that reach far beyond the health of the mother at the time of pregnancy and childbirth. These consequences can lead to her death, further morbidities or disability in the extended postpartum period (up to one year) and can negatively impact the health of her baby, the health of her other children, and the social and economic standing of her family. Except outcomes of the newborns, such consequences are poorly understood both in quality and magnitude and remain, to a large extent, without any programmatic response in low-income countries.

With limited and patchy data, maternal deaths and disabilities are considered a leading contributor to the burden of disease among women. Maternal conditions were second only to HIV/AIDS in terms of women's deaths worldwide and third in terms of disability-adjusted life-years (DALYs) for women aged 15-44 years, based on the 2005 global burden of disease estimates. More specifically, maternal conditions contributed to 2.7% of deaths among women worldwide and 12% of deaths among women aged 15-44 years. In the South-East Asian region, maternal conditions are the leading cause of women's death and responsible for 14% of deaths among women aged 15-44 years (2). As the impact of maternal deaths and disabilities is additive, it is anticipated that, with more complete data, there would be an even greater impact of the burden of maternal ill-health with concomitant economic impact on the country.

This special issue of JHPN aims to address this information vacuum about maternal morbidities and disabilities and their consequences based on findings of research from rural areas in Bangladesh and Rajasthan in India.

## THE INFORMATION VACUUM

The existing maternal health literature focuses primarily on maternal death: more than 275,000 women are estimated to die each year in pregnancy and childbirth worldwide (3-5). One known consequence of maternal death is increased mortality of the baby—stillbirth or death of the newborn (6).

While the estimates of maternal mortality and its consequences are built on relatively limited data, women who suffer from direct obstetric complications that kill—obstructed or prolonged labour, puerperal sepsis, septic abortion, severe pre-eclampsia and eclampsia, and postpartum haemorrhage—are estimated to be far higher in number yet less well-documented. The global estimates range from 15% of pregnant women suffering from complications—about 20 million women annually (7,8)—to 1-2% in resource-poor settings when the definition is restricted to the most severe morbidities (9,10).

Even less is known about the numbers and description of the consequences women may suffer as a result of pregnancy and childbirth and the life-threatening obstetric complications (11-13). These consequences—maternal morbidities or disabilities—are estimated to affect 15-20 million women worldwide each year (14). Assumed to be directly or indirectly related to difficult obstetric events, these morbidities/disabilities include conditions, such as uterine prolapse, stress incontinence, hypertension, haemorrhoids, perineal tears, urinary tract infections, severe anaemia, depression, fistula, and ectopic pregnancy.

Beyond the acute obstetric complications and potential for consequent morbidities and disabilities—either physical or mental or both—it is assumed that the health of women during pregnancy or childbirth further impacts the health and develop­ment of the next generation and the well-being of the family—both economically and socially—through impoverishment, violence, stigmatization, isolation, divorce, and remarriage. Reports from Burkina Faso tell of secondary consequences for women and their families up to a year following a severe obstetric complication, including excess mortality and mental health problems of the women (15) plus loss of physical strength, family stability, community status, and impoverishment. Such reports extend the meaning of loss beyond that quantified in measures, such as the maternal mortality ratio or DALYs (16).

As with the health of girls and women across and within countries more generally, the health of women during pregnancy and childbirth is highly affected by the social and economic factors, including education, household wealth, and the place of residence. Typically, those living in wealthier households, having higher education, or living in urban areas, have lower levels of mortality and higher use of healthcare services than their poorer, less-educated, or rural counterparts (1,17-19). What is less understood is whether these same determinants drive action and better health when a woman faces other consequences of pregnancy or childbirth—the short-term morbidities or chronic disabilities, such as postpartum depression or social consequences, such as violence.

## FUELLING THE INFORMATION VACUUM

Two major factors contribute to the information vacuum surrounding maternal ill-health—(a) the inconsistent use of terminologies to describe maternal morbidities and disabilities, and their consequences and (b) the methods used for ascertaining these quantitatively.

### Inconsistent Terminology

The inconsistent use of terminologies to describe various maternal morbidities and disabilities is a major source of confusion in interpreting the available literature. In this series, the obstetric complications that can kill are part of a wider group of morbidities suffered during the antenatal, natal or postpartum periods that we call acute maternal morbidities. Those that affect women and their families in the longer-term are called postpartum maternal morbidities and disabilities. The following section provides a general review of terms used in the literature on various conditions of maternal morbidity.

#### Defining Maternal Morbidity

***Maternal morbidity*** is an overarching term that refers to any physical or mental illness or disability directly related to pregnancy and/or childbirth. These are not necessarily life-threatening but can have a significant impact on the quality of life.

***Acute maternal morbidities*** include various terms, such as ‘obstetric complications’, ‘maternal complications’, ‘absolute maternal indications’ (AMIs), ‘severe acute maternal morbidities’ (SAMMs), and ‘near-miss’ and typically refers to acute problems suffered during pregnancy through the standard postpartum period of 42 days.

*Obstetric or maternal complications* are acute conditions that may directly cause maternal deaths. According to the United Nations Children's Fund/WHO/United Nations Population Fund (1997) ‘complicated cases’ include antepartum or postpartum haemorrhage, prolonged or obstructed labour, postpartum sepsis, complications of abortion, pre-eclampsia/eclampsia, ectopic pregnancy, and ruptured uterus (8). Anaemia, malaria, tuberculosis, and other pre-existing conditions that may complicate delivery are considered indirect obstetric complications. Rarely are the definitions for these terms for obstetric complications—direct or indirect—more specified.

*Severe obstetric complications* have been defined variously based on the criteria of disease, management and/or organ failure/dysfunction as follows:

*Absolute maternal indications (AMIs)* are life-threatening or severe obstetric complications requiring a specific major obstetric intervention which can be verified through records of health services. AMIs reflect conditions that, without intervention, have a high probability of causing maternal death during childbirth or sequelae including the following (20):

- Severe antepartum haemorrhage
- Placenta praevia and abruptio placentae
- Severe postpartum haemorrhage requiring surgical intervention
- Foetopelvic disproportion (pre-rupture and uterine rupture)
- Shoulder or transverse lie

*Severe acute maternal morbidities (SAMMs)* include complications that are ‘absolutely’ life-threatening using concepts of organ failure and lifesaving surgery—such that women who experience these problems are unlikely to survive if they do not receive care in a hospital (9).

*Near-miss* is defined by the WHO as “a woman who nearly died but survived a complication that occurred during pregnancy, childbirth or within 42 days of termination of pregnancy” (21), or to put more simply, “… women are considered near-miss cases when they survive life-threatening conditions (i.e. organ dysfunction)” (22). The criteria to determine a near-miss condition are based on organ-system dysfunction or failure versus disease-specific or intervention-specific criteria as the organ-based criteria are found to be more specific in identifying real severe acute maternal morbidity cases (10). The organ-system-based criteria include cardiovascular, respiratory, renal, coagulation/haematological, hepatic, neurological and uterine dysfunction (22).

***Postpartum maternal morbidities and disabilities*** are the long-term physical or mental consequences resulting from pregnancy, childbirth, acute maternal morbidities, or the management thereof, and most often referred to as long-term chronic morbidities and other problems experienced postpartum (23).

***Chronic morbidities*** are conditions caused by the birthing process and are not life-threatening but greatly impair the quality of life, such as fistula, uterine prolapse, and dyspareunia.

*Milder disabilities* are also called postpartum maternal morbidities and include urinary incontinence, hernias, haemorrhoids, breast problems, and postpartum depression.

### Methodological Loopholes and the Way Out

#### What Is Valid Reporting of Morbidities and Disabilities?

Capturing maternal morbidity and its consequences where women do not usually use skilled care providers or facilities for delivery has been difficult. Under such circumstances, women's self-report in response to survey questionnaire or interviews by community-based health workers has been the primary means to obtain data.

***Limitation in using self-reported complications:*** Results of studies conducted in the mid-1990s demonstrate that the reliability of self-reported complications based on a woman's recall is poor compared to medical records, even if the woman suffered from a life-threatening complication (24-27).

***Assessment by community-based healthcare providers:*** Many studies have worked with community-based healthcare providers to assess acute maternal morbidities (28-35). These community-based care providers most likely had differing levels of training, supervision, and equipment to diagnose maternal complications. The reliability and validity of these assessments and, obviously, comparability, are unclear. Even with the assessment by community workers followed up by skilled care providers in the community, the type of measurement, done by whom, and timing of the assessment and of the complications, can vary widely. For example, in a review of maternal morbidity in India and Bangladesh, puerperal sepsis identified by community workers was defined as fever lasting for three or more days, up to two weeks or up to six weeks postpartum in different studies (28,30,34).

***Assessment of gold standard—skilled providers in facility:*** The ‘gold’ standard for the diagnosis of morbidity remains assessment by skilled care providers at a health facility. Ronsmans argues that, using facility-based diagnoses by skilled care providers based on organ failure and lifesaving surgery to determine SAMMs, one can estimate the population levels of severe maternal morbidity—as women with such problems will die if not managed in such facilities (9). She acknowledges that the management criteria continue to be only partially reliable across settings because of the human element but that the criteria for lifesaving surgery are more standardized, and comparable population-based data are becoming increasingly available.

A recent Maternal Mortality and Morbidity Classifications Working Group of the WHO outlined criteria to determine severe obstetric morbidity (near-miss) that is more limited than SAMMs, i.e. women presenting with features of organ dysfunction. They have also developed tools and outlined a process of gathering data to improve comparability across studies (22).

## STUDIES OF MATERNAL MORBIDITIES AND DISABILITIES IN THIS SPECIAL ISSUE OF JHPN

**Fig. 1.**
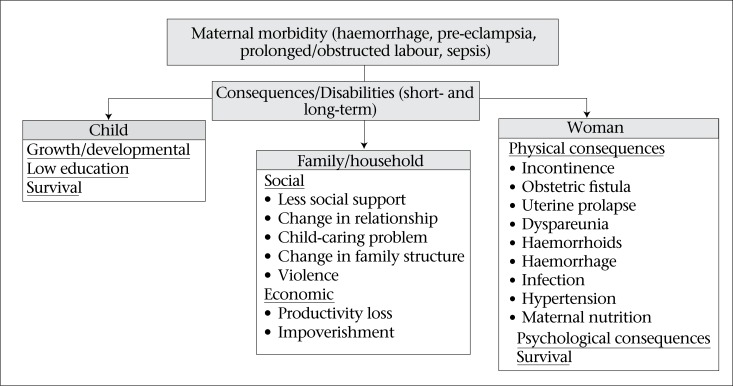
Conceptual framework for the study of maternal morbidity and its consequences

This special issue of the Journal aims to respond to the major gaps in knowledge with studies on acute and postpartum maternal morbidities and disabilities from Matlab in Bangladesh and Rajasthan in India. A conceptual framework for this work is depicted in Figure 1. The studies specifically report the following:

- The level of severe and less-severe acute maternal morbidities during pregnancy, childbirth, and postpartum (42 days) in rural Bangladesh and Rajasthan in India
- The consequences of maternal morbidity (postpartum morbidities and disabilities experienced up to nine weeks in Bangladesh or 12 months in India, including depression (for the women), and death or developmental delays (for her newborn)
- The longer-term impact on women and families—mentally, socially, and economically (Bangladesh) and deaths of children or mothers up to one year postpartum (India).

### Hypotheses

The hypotheses underlying the studies of maternal morbidities, disabilities, and consequences include the following:

- Women who suffer from severe acute maternal morbidities are at risk of suffering from long-term consequences (e.g. physical, social and mental consequences) or death compared to those with vaginal deliveries with no complications.
- Women who suffer from moderate and severe acute maternal morbidities and those who die are at higher risk of suffering poor pregnancy outcomes (e.g. stillbirths, neonatal death, and infant death) compared to those with vaginal deliveries with no complications.
- A child of a mother suffering from long-term consequences of severe acute maternal morbidities is at higher risk of death and poorer development than those of women without such consequences.
- Families of women who have suffered from severe acute maternal morbidities (and/or poor pregnancy outcomes) are at higher risk of dissolution, violence, and/or impoverishment.

### Studies and the resulting papers

Driven by the above hypotheses, three types of studies were conducted, each with qualitative and quantitative components. The papers detailing the findings of each study type are listed below.

1. Examination of the incidence of short- and long-term morbidities and physical disabilities of women with severe and less-severe acute maternal morbidities (obstetric complications) and those with normal vaginal deliveries in rural Bangladesh and India:
  - Profile of maternal and foetal complications during labour and delivery among women giving birth in hospitals in Matlab and Chandpur, Bangladesh (Huda *et al*.)
  - Occurrence and determinants of maternal postpartum morbidities and disabilities among women in Matlab, Bangladesh (Ferdous *et al*.)
  - Early postpartum maternal morbidity among rural women of Rajasthan, India: a community-based study (Iyengar)
  - Consequences of maternal complications on women's lives in the first postpartum year: a prospective cohort study (Iyengar *et al*.).
  Findings regarding morbidity or disability relating to abortion or complications of abortion are not included in the studies reported in this special issue of the Journal.
2. Determination of the outcomes of the newborn (death and developmental delays) as related to maternal morbidity/mortality:
  - Consequences of maternal complications on women's lives in the first postpartum year: a prospective cohort study (Iyengar *et al.*)
  - Profile of maternal and foetal complications during labour and delivery among women giving birth in hospitals in Matlab and Chandpur, Bangladesh (Huda *et al.*)
  - Association of postpartum maternal morbidities with children's mental, psychomotor and language development in rural Bangladesh (Hamadani *et al.*).
3. Documentation of the psychological, social and economic impacts of maternal ill-health and death (maternal and perinatal) on women and other members living in the family unit:
  - Obstetric complications and psychological well-being: Bangladeshi women's experiences with pregnancy and childbirth (Gausia *et al.*)
  - An examination of women experiencing obstetric complications requiring emergency care: perceptions and sociocultural consequences of caesarean sections in Bangladesh (Khan *et al.*)
  - Violence against women with chronic maternal disabilities in rural Bangladesh (Naved *et al.*)
  - Costs of maternal health complications in Bangladesh (Hoque *et al.*)
  - Early postpartum maternal morbidity among rural women of Rajasthan, India: a community-based study (Iyengar).

### Study sites

**Fig. 2.**
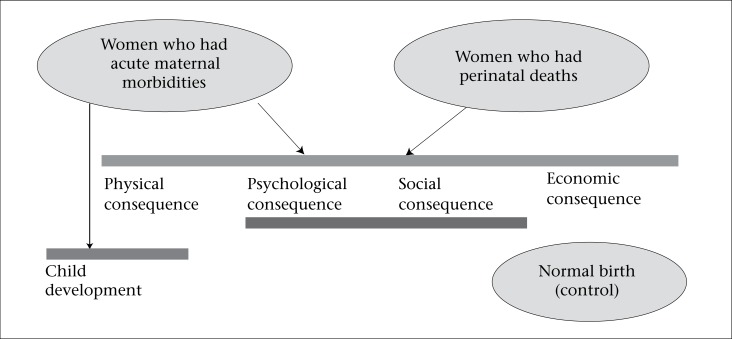
Matlab prospective study

In Bangladesh, the icddr,b's community data from the Matlab intervention area with its population of 110,000 plus facility data from Matlab and Chandpur district town provide a unique opportunity to capture the levels of maternal mortality, morbidities, and disabilities while tracing women and families who have suffered and linking them to changes in familial, social and economic status over time. This is done through secondary analysis of the existing data dating back 30 years plus prospective data collected over 24 months starting in 2007. In the prospective study, only those women who had a care provider's diagnosis of morbidity were included (Fig. 2 for study design). Ninety-two percent of women who had a hospital admission, a live- or stillbirth outcome, and who had records of diagnosis by care provider, were traced representing 36% of all pregnancies in the Matlab area with icddr,b interventions over the life of the project. All other women delivered at home, in subcentres (health centres), in sites beyond Matlab/Chandpur, or their records could not be traced. We assume that women with a hospital delivery were those with the most serious acute morbidities; those who died during this period (12 maternal deaths) were also known and are reported in the paper of Huda *et al*.

In India, Action Research and Training for Health (ARTH), a non-profit organization based in Udaipur, provides data on maternal morbidities and their consequences from a 36-month prospective study (starting in 2007) on a rural tribal population of 58,000 in southern Rajasthan. Given that few women in the area used facilities for deliveries, morbidity data on all women with a birth in the area were collected by midwives within 2-3 days of birth during home-visits, using a checklist developed to question women on pregnancy or intrapartum complications and their consequences; they also carried out a physical examination, including measurement of haemoglobin. Follow-up visits of these women continued for 12 months postpartum. Recently-delivering women were identified by the family members or community workers [accredited social health activists (ASHAs) and village health volunteers] who were paid by ARTH for providing information on births.

### Burden of disease for maternal deaths and disabilities

Finally, we contemplated the recalculation of the burden of disease for maternal death and disabilities for Bangladesh, using the methodology of the Global Burden of Disease Study (36) with the maternal mortality, morbidity and disability data from Matlab. This exercise made clear that most maternal morbidities and disabilities were not assigned any weight in the methodology of the 2004 Updated Burden of Disease (Box), and calculations of the burden of disease for maternal conditions were and remain highly underestimated.

**Calculation of DALY for Maternal Conditions—What Is Missing?**

*Global burden of disease morbidities* for which there are DALYs include anaemia, cardiovascular conditions, obstructed labour, haemorrhage, hypertensive disorders, reproductive tract infections, and sepsis.

*Unaccounted disabilities in the global burden of disease calculation (as of 2008)* include dyspareunia, genital prolapse, haemorrhoids, mastitis, stillbirths, perineal tears, postpartum depression, urinary tract infections, and vulvar disruption.

Such an undervaluation is only secondary to the underestimation of the incidence of maternal morbidities and disabilities and to the undervaluation of women's health more generally.

## ACKNOWLEDGEMENTS

The authors are grateful to Evelyn Ford (Intern), Sushil Dasgupta (icddr,b), and Sandee Minovi (John Snow Inc.) for their assistance with this document. They also thank the United States Agency for International Development for funding this effort and for stimulating them to think beyond the conventional boundaries of the topic.

## References

[1] World Health Organization (2009). Women and health: today's evidence tomorrow's agenda.

[2] Ribeiro PS, Jacobsen KH, Mathers CD, Garcia-Moreno C (2008). Priorities for women's health from the Global Burden of Disease study. Int J Gynaecol Obstet.

[3] World Health Organization (2010). Trends in maternal mortality: 1990 to 2008.

[4] Hogan MC, Foreman KJ, Naghavi M, Ahn SY, Wang M, Makela SM (2010). Maternal mortality for 181 countries, 1980-2008: a systematic analysis of progress towards Millennium Development Goal 5. Lancet.

[5] Lozano R, Wang H, Foreman KJ, Rajaratnam JK, Naghavi M, Marcus JR (2011). Progress towards Millennium Development Goals 4 and 5 on maternal and child mortality: an updated systematic analysis. Lancet.

[6] Lawn JE, Lee AC, Kinney M, Sibley L, Carlo WA, Paul VK (2009). Two million intrapartum-related stillbirths and neonatal deaths: where, why, and what can be done?. Int J Gynaecol Obstet.

[7] World Health Organization (2009). Monitoring emergency obstetric care: a handbook.

[8] United Nations Children's Fund (1997). Guidelines for monitoring the availability and use of obstetric services.

[9] Ronsmans C (2009). Severe acute maternal morbidity in low-income countries. Best Pract Res Clin Obstet Gynaecol.

[10] Say L, Pattinson RC, Gülmezoglu AM (2004). WHO systematic review of maternal morbidity and mortality: the prevalence of severe acute maternal morbidity (near miss). Reprod Health.

[11] Koblinsky M, Conroy C, Kureshy N, Stanton ME, Jessop S (2000). Issues in programming for safe motherhood.

[12] Filippi V, Ronsmans C, Campbell OMR, Graham WJ, Mills A, Borghi J (2006). Maternal health in poor countries: the broader context and a call for action. Lancet.

[13] Ronsmans C, Graham WJ, Lancet Maternal Survival Series steering group (2006). Maternal mortality: who, when, where, and why. Lancet.

[14] Murray CJL, Lopez AD (1998). Health dimensions of sex and reproduction.

[15] Filippi V, Ganaba R, Baggaley RF, Marshall T, Storeng KT, Sombié I (2007). Health of women after severe obstetric complications in Burkina Faso: a longitudinal study. Lancet.

[16] Storeng KT, Murray SF, Akoum MS, Ouattara F, Filippi V (2010). Beyond body counts: a qualitative study of lives and loss in Burkina Faso after ‘near-miss’ obstetric complications. Soc Sci Med.

[17] Anwar I, Kalim N, Koblinsky M (2009). Quality of obstetric care in public-sector facilities and constraints to implementing emergency obstetric care services: evidence from high- and low-performing districts of Bangladesh. J Health Popul Nutr.

[18] Chowdhury ME, Ahmed A, Kalim N, Koblinsky M (2009). Causes of maternal mortality decline in Matlab, Bangladesh. J Health Popul Nutr.

[19] Wang W, Alva S, Wang S, Fort A (2011). Levels and trends in the use of maternal health services in developing countries.

[20] Belghiti A, De Brouwere V, Kegels G, Van Lerberghe W (1998). Monitoring unmet obstetric need at district level in Morocco. Trop Med Int Health.

[21] Say L, Souza JP, Pattinson RC, WHO working group on Maternal Mortality and Morbidity classifications (2009). Maternal near miss–towards a standard tool for monitoring quality of maternal health care. Best Pract Res Clin Obstet Gynaecol.

[22] World Health Organization (2011). Evaluating the quality of care for severe pregnancy complications: the WHO near-miss approach for maternal health.

[23] Ashford L (2002). Hidden suffering: disabilities from pregnancy and childbirth in less developed countries. Policy brief.

[24] Filippi V, Ronsmans C, Gandaho T, Graham W, Alihonou E, Santos P (2000). Women's reports of severe (near-miss) obstetric complications in Benin. Stud Family Plann.

[25] Ronsmans C, Achadi E, Cohen S, Zazri A (1997). Women's recall of obstetric complications in South Kalimantan, Indonesia. Stud Fam Plann.

[26] Seoane G, Castrillo M, O'Rourke K (1998). A validation study of maternal self reports of obstetrical complications: implications for health surveys. Int J Gynecol Obstet.

[27] Stewart MK, Festin M (1995). Validation study of women's reporting and recall of major obstetric complications treated at the Philippine General Hospital. Int J Gynaecol Obstet.

[28] Bang RA, Bang AT, Reddy MH, Deshmukh MD, Baitule SB, Filippi V (2004). Maternal morbidity during labour and the puerperium in rural homes and the need for medical attention: a prospective observational study in Gadchiroli, India. BJOG.

[29] Fronczak N (1996). Early maternal morbidity and utilization of delivery services by urban slum women of Dhaka.

[30] Goodburn EA, Chowdhury M, Gazi R, Marshall T, Graham W, Karim F (1994). An investigation into the na­ture and determinants of maternal morbidity related to delivery and the puerperium in rural Bangladesh. Dhaka: Research and Evaluation Division, BRAC. Health Studies.

[31] Kusiako T, Ronsmans C, Van der Paal L (2000). Perinatal mortality attributable to complications of childbirth in Matlab, Bangladesh. Bull World Health Organ.

[32] Maine D, Akalin MZ, Chakraborty J, de Francisco A, Strong M (1996). Why did maternal mortality decline in Matlab?. Stud Fam Plann.

[33] Razzaque A, Da Vanzo J, Rahman M, Gausia K, Hale L, Khan MA (2005). Pregnancy spacing and maternal morbidity in Matlab, Bangladesh. Int J Gynaecol Obstet.

[34] Uzma A, Underwood P, Atkinson D, Thackrah R (1999). Postpartum health in a Dhaka slum. Soc Sci Med.

[35] Vanneste AM, Ronsmans C, Chakraborty J, De Francisco A (2000). Prenatal screening in rural Bangladesh: from prediction to care. Health Policy Plan.

[36] World Health Organization (2008). The global burden of disease: 2004 update.

